# Augmenting Autophagy to Treat Acute Kidney Injury during Endotoxemia in Mice

**DOI:** 10.1371/journal.pone.0069520

**Published:** 2013-07-30

**Authors:** Gina M. Howell, Hernando Gomez, Richard D. Collage, Patricia Loughran, Xianghong Zhang, Daniel A. Escobar, Timothy R. Billiar, Brian S. Zuckerbraun, Matthew R. Rosengart

**Affiliations:** Department of Surgery, University of Pittsburgh, Pittsburgh, Pennsylvania, United States of America; University of Kentucky, United States of America

## Abstract

**Objective:**

To determine that 1) an age-dependent loss of inducible autophagy underlies the failure to recover from AKI in older, adult animals during endotoxemia, and 2) pharmacologic induction of autophagy, even after established endotoxemia, is of therapeutic utility in facilitating renal recovery in aged mice.

**Design:**

Murine model of endotoxemia and cecal ligation and puncture (CLP) induced acute kidney injury (AKI).

**Setting:**

Academic research laboratory.

**Subjects:**

C57Bl/6 mice of 8 (young) and 45 (adult) weeks of age.

**Intervention:**

Lipopolysaccharide (1.5 mg/kg), Temsirolimus (5 mg/kg), AICAR (100 mg/kg). Measurements and Main Results: Herein we report that diminished autophagy underlies the failure to recover renal function in older adult mice utilizing a murine model of LPS-induced AKI. The administration of the mTOR inhibitor temsirolimus, even after established endotoxemia, induced autophagy and protected against the development of AKI.

**Conclusions:**

These novel results demonstrate a role for autophagy in the context of LPS-induced AKI and support further investigation into like interventions that have potential to alter the natural history of disease.

## Introduction

Globally, the population is aging, and the incidence, morbidity and mortality of sepsis increase with advancing age [1], [2]. With the onset of organ failure, sepsis progresses to severe sepsis, and the kidney is arguably the most commonly affected organ. Advanced age is an independent risk factor for non-recovery of renal function after AKI [3]. Once established, therapy is supportive, while renal function, hopefully, returns. The costs to both patient and society are substantial [4].

Autophagy, an evolutionarily conserved process of cellular self-digestion, may be important in the *recovery* from AKI. Constitutive basal autophagy maintains homeostasis by regulating biomass quantity, quality and distribution. But autophagy can also be induced in response to multiple forms of stress, including sepsis, where it is largely thought to promote cell survival [5]. Recent literature suggests a critical cytoprotective role for autophagy in both toxin-mediated and ischemia-reperfusion-induced AKI [6]. Autophagic mechanisms directed at removal of damaged mitochondria, or *mitophagy*, are considered of particular importance in protecting against AKI, which primarily affects the mitochondria-rich proximal tubule cells [6]. However, evidence that harnessing this autophagic machinery to treat established AKI is lacking.

Experimental animal models suggest that autophagy is diminished in the aging kidney, and that proximal tubule cells fail to induce autophagy during ischemic stress that correlates with the development of age-dependent AKI [7]. Given these observations, we hypothesized that 1) an age-dependent loss of inducible autophagy underlies the failure to recover from AKI in adult animals during endotoxemia, and 2) pharmacologic induction of autophagy, even after established endotoxemia, is of therapeutic utility in facilitating renal recovery.

## Materials and Methods

### Ethics Statement

All experiments were performed in accordance with the National Institutes of Health guidelines under protocols approved by the Institutional Animal Care and Use Committee of the University of Pittsburgh (protocol # 1006391B). All surgery was performed under halothane inhalational anesthesia, and all efforts were made to minimize suffering.

### Mice

Male C57BL/6 mice (Jackson Laboratories, Bar Harbor, ME) ∼8 weeks (young) and ∼45 weeks (adult) mice were utilized for all *in vivo* studies. This 45 week “middle-aged” group was preferentially chosen as they exhibit known biomarkers of aging and are more susceptible to injury without excessive mortality, a potential competing risk for our primary outcome of acute kidney injury [8].

### In vivo RNAi

Autophagy was inhibited using in vivo RNAi of VPS34 as previously performed; this technique effectively and specifically inhibits the expression of the targeted protein of interest [9], [10]. Mice were administered VPS34 or scrambled, non-target siRNA (6 mg/kg) by hydrodynamic tail vein injection delivered in (animal mass/10) mL lactated ringers as previously performed [9]. After 72 hours mice were randomly allocated to each experimental condition.

### Endotoxemia

Ultra Pure LPS (Escherichia coli 0111:B4) from LIST Biologicals (Campbell, CA) was dissolved in sterile normal saline and injected intraperitoneally (1.5 mg/kg). At various time points after LPS, mice were euthanized, blood was isolated by cardiac puncture, and the kidneys were harvested.

### Cecal ligation and puncture

We performed cecal ligation and puncture (CLP) as previously described, using a single 21-gauge puncture, a model we have optimized to enable the evaluation of cellular/organ biology and physiology [11]. Sham animals underwent anesthesia and laparotomy with bowel manipulation. All mice received volume resuscitation with 0.9% saline (2 ml/kg SQ).

### Induction of Autophagy

Temsirolimus (TORISEL®, Wyeth Pharmaceuticals, Madison, NJ) is provided as a concentrated injectable form, which must first be mixed with DILUENT for TORISEL®, a sterile non-aqueous solution that is supplied with the active drug as a kit. TORISEL was administered by tail vein injection (5 mg/kg). Control animals received equivolume DILUENT control vehicle. Injections were administered 2 hours before or after LPS. Alternatively we used AICAR (5-Aminoimidazole-4-carboxamide 1-β-D-ribofuranoside, Acadesine, N^1^-(β-D-Ribofuranosyl)-5-aminoimidazole-4-carboxamide) (Sigma-Aldrich, St. Louis, MO) administered at 100 mg/kg by intraperitoneal injection 24 hours prior to CLP [12], [13], [14], [15].

### Cellular protein extraction

Total cellular lysate was extracted at 4°C in 500 µL of lysis buffer [11]. Protein concentration was determined using a bicinchoninic acid protein assay (Pierce, Rockford, IL).

### Western blotting

Total cellular lysate was electrophoresed in either an 8% or 15% SDS-PAGE gels and then transferred to a Hybond-enhanced chemiluminescence nitrocellulose membrane (Amersham Pharmacia Biotech, Piscataway, NJ) [11]. The membrane was blocked for 1 hour at room temperature with 5% milk and incubated with primary antibody against LC3b for 16 hours at 4°C. Blots were then incubated in a horseradish peroxidase-conjugated secondary antibody at room temperature for 1 hour. The blot was developed using Luminata^TM^ Crescendo Western HRP Substrate (Millipore, Billerica, MA), and was exposed on KAR-5 film (Eastman Kodak, Rochester, NY). Densitometry was performed by the NIH image program (National Institutes of Health, Bethesda, MD) to quantitate optical density. Antibodies for total LC3b, p-Ser^2448^ mTOR, and tubulin were obtained from Abcam (Cambridge, MA).

### Immunohistochemistry

Kidneys were flushed with PBS and then perfused with 2% paraformaldehyde. After 2 h fixation, samples were transferred to 30% sucrose for 24 h with a total of three sucrose changes. The samples were cryopreserved in liquid nitrogen cooled 2-methylbutane and stored at −80°C until sectioning. Cryopreserved tissues were sectioned to 6 mm thickness and incubated with 2% bovine serum albumin (BSA) in PBS for 1 h, followed by 5 washes with PBS+0.5% BSA (PBB). This was followed by an overnight incubation of samples with anti-LC3-I/II antibody in PBS+5% BSA (Novus, 5 ug/ml) in the presence of Triton X-100 at 0.1% at 4C. The slides were washed with 0.5% BSA, followed by a 1 hour incubation with a Cy3 secondary antibody (goat anti-rabbit, 1:1000, Jackson ImmunoResearch Laboratories) plus AlexaFluor-488 phallodin (1:250, Invitrogen). The slides were rinsed, and HOECHST dye (1 mg/100 ml bisbenzimide) was applied for 30 s. The slides were rinsed with PBS and coverslipped with gelvatol, a water-soluble mounting media. Slides were imaged with an Olympus Fluoview 1000 confocal scanning microscope (Olympus, Melville, NY). Imaging conditions were maintained at identical settings within each antibody-labeling experiment with original gating performed using the negative control. Quantification was performed using Metamorph to determine the mean fluorescent intensity (MFI) of punctate LC3b adjusted for actin MFI (Molecular Devices, Sunnyvale, CA).

### Electron Microscopy

Kidneys were flushed with PBS and subsequently perfused and fixed with 2% glutaraldehyde in 0.1 mol/L phosphate buffer (pH 7.4), followed by 1% OsO_4_. After dehydration with graded alcohols, the samples were embedded in epoxy resin (Epon). Thin sections (70 nm) were then cut by a microtome (Leica Ultracut R), mounted on copper grids and post-stained with 2% uranyl acetate and 1% lead citrate, dried, and analyzed using a JEM 1011CX electron microscope (JEOL, Peabody, MA). Images were acquired digitally from a randomly selected pool of 10 to 15 fields for each experimental condition. A semiquantitative analysis of autophagosome density was performed using the methodology of Swanlund et al. [16].

### Renal function parameters

Renal function was determined by assaying serum for blood urea nitrogen (BUN) and cystatin C, respectively using the DRI-CHEM 4000 Chemistry Analyzer (Heska, Loveland, CO) and an enzyme immunoassay kit (R&D, Minneapolis, MN). Cystatin C has emerged as a more precise marker of glomerular filtration rate and has been validated in both human and murine studies [17], [18].

### Statistics

For all studies, one investigator performed the surgical experimentation and collected the samples. A separate individual administered the temsirolimus or control diluent without prior knowledge of the surgical treatment. The data were then analyzed by an investigator blinded to the specific treatment. A total of at least 8 mice per experimental condition were used to ensure a beta = 0.2, assuming a two-sided alpha = 0.05. Statistical analyses were performed using Stata 12SE software (College Station, TX). Data are reported as means + SEM. Mann-Whitney was utilized for comparison between groups. P values less than 0.05 were considered significant.

## Results and Discussion

**Figure 1:**
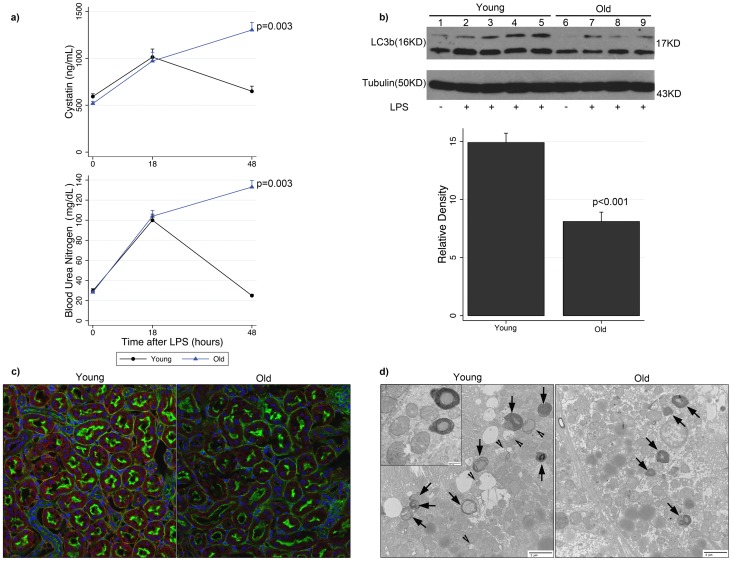
Adult mice exhibit non-recovery of renal function and diminished autophagy during endotoxemia. (a) Time course plots for serum BUN and cystatin concentrations after LPS comparing adult and young mice (n = 8–11 mice per group per timepoint). (b) The effects of LPS on autophagy in the renal cortices of adult and young mice were assessed by immunoblot for LC3b (16KD). Representative blot is shown at 48 h timepoint after LPS from n = 4 experiments (8–9 mice per experiment). Corresponding densitometry compares total LC3b of older aged adult and younger animals exposed to LPS. (c) Immunofluorescent microscopy (20X) of renal cortex in adult and young mice harvested at 48 h after LPS. LC3 (Cy3, red), actin (Alexa488, green), nucleus (Hoechst, blue). Representative images of n = 4 experiments (8 mice per experiment). (d) Transmission electron microscopy (10^5^X) of renal cortex of young and adult mice 48 h after endotoxemia. Inset (5×10^5^X). Representative images of n = 4 experiments (8 mice per experiment). arrowheads, autophagosomes; arrows, autophagolysosomes. Data are means ± s.e.m.; rank sum test.

As shown in Figure 1a, adult mice, by comparison to their younger counterparts, exhibited non-recovery of renal function after LPS administration. Both groups have similar BUN and cystatin concentrations at baseline and 18 hours after LPS, the latter indicating comparable susceptibility to AKI. By 48 hours, however, young mice demonstrated normalization of renal function, whereas adult mice exhibited persistent elevations in both BUN (25 vs. 133 mg/dL, p = 0.003) and cystatin (649 vs. 1302 ng/mL, p = 0.003).

The impairment in renal recovery observed in adult mice correlated with reduced renal autophagy as assessed by three independent methods: immunoblot, immunofluorescence, and electron microscopy (EM). Representative immunoblot and corresponding densitometry show significantly less LC3b expression in adult animals than in younger animals 48 hours after LPS (p<0.001) (Fig. 1b). Young animals, by contrast to adult animals, exhibited more immunofluorescence and a more pronounced punctate staining pattern for LC3 (MFI: 0.062 vs. 0.003, p = 0.03), the latter indicating the association of the conjugated form of LC3 (LC3b) with the autophagosomal membrane (Fig. 1c). EM illustrates numerous multi-membranous autophagosomes and autophagolysosomes in the renal cortex of younger animals, which are notably reduced in adult animals, which exhibit swollen, damaged mitochondria lacking prototypical architectural features (Fig. 1d): proportion of cytoplasmic area 0.06 vs. 0.029, p = 0.005. We did not observe notable differences in basal (i.e. without LPS) autophagy between young and adult animals (Fig. 1b; immunofluorescence and electron microscopy data not shown).

**Figure 2:**
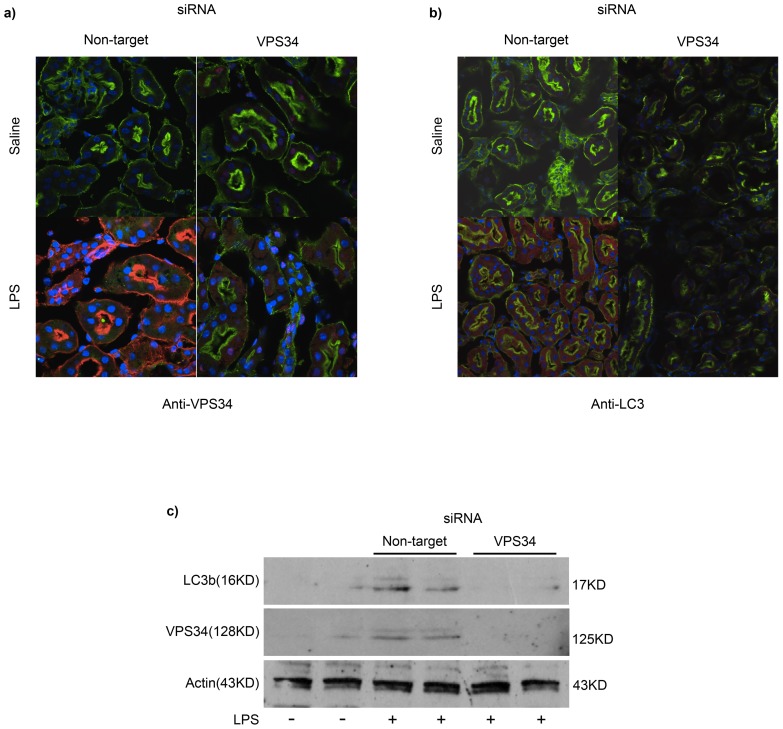
Inhibition of VPS34 attenuates autophagy and inhibits recovery of renal function in young mice during endotoxemia. Immunofluorescent microscopy (20X) of renal cortex harvested at 48 hours after LPS in young mice from 4 experimental groups: NT siRNA and saline control, VPS34 siRNA and saline control, NT siRNA and LPS, and VPS34 siRNA and LPS. (a) VPS34 (Cy3, red), actin (Alexa488, green), nucleus (Hoechst, blue). Representative images of n = 4 experiments (4 mice per experiment). (b) LC3 (Cy3, red), actin (Alexa488, green), nucleus (Hoechst, blue). Representative images of n = 4 experiments (4 mice per experiment). (c) Immunoblot analysis of renal cortex lysate detecting LC3b (16KD) in untreated mice (lanes 1 and 2) and mice treated with Non-target siRNA and LPS (lanes 3 and 4) or VPS34 siRNA and LPS (lanes 5 and 6). Representative blot is shown at 48 h timepoint after LPS from n = 2 experiments (6 mice per experiment).

We hypothesized that inhibiting autophagy would render young animals ‘adult’ and interfere with recovery from AKI. VPS34, an evolutionarily conserved class III phosphoinosititde 3-kinase, is an indispensible upstream regulator of autophagy [10]. We have previously used RNAi to inhibit the *in vivo* expression of VPS34 as means to regulate autophagy *in vivo* [9], [10]. As shown in Figure 2a, by comparison to non-target, scrambled siRNA (RNAi^NT^), VPS34 siRNA (RNAi^VPS34^) effectively inhibited the induced expression of VPS34 in renal tubular cells during endotoxemia (Fig. 2a.), which correlated with attenuated autophagy, as evidenced by reduced punctate LC3 expression (Fig. 2b). Representative immunoblot shows significantly less LC3b expression in RNAi^VPS34^ by comparison to RNAi^NT^ animals 48 hours after LPS (Fig. 2c). RNAi^VPS34^ mice, by comparison to RNAi^NT^ mice, failed to recover from AKI, as characterized by persistently elevated BUN (29 vs. 133 mg/dL, p = 0.02) and cystatin (636 vs. 1367 ng/mL, p = 0.02) concentrations 48 hours after LPS. Thus, inhibiting autophagy in young mice produced a temporal pattern of AKI similar to that of aged, adult mice.

These data suggest that loss of autophagy in the kidney may underlie the failure to recover from septic AKI in aged mice. Thus, restoring autophagic activity in older animals could potentially facilitate renal recovery. The mammalian target of rapamycin (mTOR) serves as a negative regulator of autophagy, and as a consequence, initiation of autophagy is largely dependent on release of mTOR inhibition. Studies have explored the use of pharmacologic mTOR inhibitors, such as rapamycin (sirolimus) and its analogues, such as temsirolimus, which potently inhibit downstream signaling from mTOR proteins and regulate a number of cellular processes including autophagy [19], [20]. Evidence is emerging that rapamycin may have global anti-aging potential with extension of lifespan in animal models [21]. Further, in a murine cecal ligation and puncture (CLP) model of sepsis, rapamycin increased autophagy and restored cardiac contractility [22].

**Figure 3:**
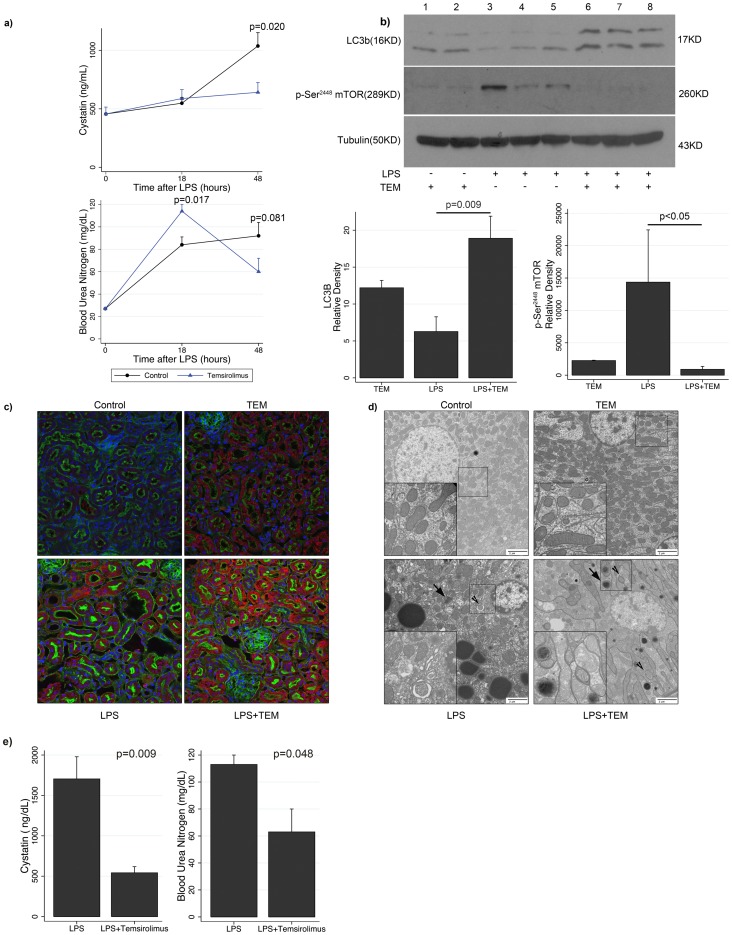
Temsirolimus improves renal function and increases renal autophagy in adult mice during endotoxemia. (a) Time course plots for serum cystatin and blood urea nitrogen (BUN) concentrations after LPS in adult mice receiving either temsirolimus or diluent control (n = 8–11 mice per group per timepoint). (b) Immunoblot analysis of renal cortex lysate detecting LC3b (16KD) and p-Ser^2448^ mTOR (289KD) in mice treated with temsirolimus (lanes 1 and 2), diluent control and LPS (lanes 3–5), or temsirolimus and LPS (lanes 6–8). Representative blot is shown at 48 h timepoint after LPS from n = 4 experiments (8 mice per experiment). Graphs summarizing corresponding density of LC3b and p-Ser^2448^ mTOR for each experimental condition. (c) Immunofluorescent microscopy (20X) of renal cortex harvested at 48 hours after LPS in 4 experimental groups: diluent control, temsirolimus, diluent control and LPS, and temsirolimus and LPS. LC3 (Cy3, red), actin (Alexa488, green), nucleus (Hoechst, blue). Representative images of n = 4 experiments (8 mice per experiment). (d) Transmission electron microscopy (10^5^X) of renal cortex harvested at 48 hours after LPS in 4 experimental groups. Inset (5×10^5^X) Representative images of n = 4 experiments (8 mice per experiment). arrowheads, autophagosomes; arrows, autophagolysosomes. (e) Serum cystatin and blood urea nitrogen (BUN) concentrations in adult mice receiving either temsirolimus or diluent control after endotoxemia (n = 8–11 mice per group per timepoint). Abbreviations: TEM, temsirolimus. Data are means ± s.e.m.; rank sum test.

We observed that mTOR inhibition using the rapalogue temsirolimus, augmented autophagy and protected against the development of AKI in the context of endotoxemia. Aged mice receiving temsirolimus 2 hours prior to LPS had significantly lower cystatin (641 vs. 1037 ng/mL, p = 0.02) and a trend toward lower BUN (60 vs. 92 mg/dL, p = 0.081) concentrations at 48 hours, compared with animals that received diluent control prior to LPS (Fig. 3a). However, aged adult mice administered temsirolimus prior to LPS had higher serum BUN concentration at 18 hours than those that received diluent control prior to LPS (114 vs. 84 mg/dL, p = 0.017), affirming that both groups experienced similar insult in the acute phase. Mice receiving temsirolimus alone did not demonstrate derangements in these renal parameters (data not shown).

All three methods utilized to detect the presence of autophagy, demonstrated that temsirolimus induced autophagy (Fig. 3b–d). Immunoblot and corresponding densitometry illustrate significantly higher expression of LC3b in endotoxemic animals receiving temsirolimus versus those that did not (p = 0.009) (Fig. 3b). These data are corroborated by immunofluorescent images (Fig. 3c). By contrast to control animals, temsirolimus alone significantly induced autophagy (MFI: 0.0 vs. 0.04, p = 0.04). We observed a more prominent overall immunofluorescence and punctate staining pattern for LC3 in animals receiving temsirolimus followed by LPS compared to other experimental groups. Similarly, EM images highlight an increased number of autophagosomes in the renal cortex of mice receiving temsirolimus versus diluent control (Fig. 3d). The ability of temsirolimus to increase renal autophagy in the setting of endotoxemia is also noted by the presence of quantitatively more autophagosomes (proportion of cytoplasmic area 0.022 vs. 0.011, p = 0.03), which was accompanied by less mitochondrial architectural damage in this group (Fig. 3d): Endotoxemia was associated with activation of renal cortical mTOR, as evidenced by increased p-Ser^2448^ mTOR: Densitometry: 2253 vs. 14362, p = 0.08 (Fig. 3b). The administration of temsirolimus during endotoxemia inhibited mTOR activation, as evidenced by reduced p-Ser^2448^ mTOR: Densitometry 14362 vs. 897, p<0.05 (Fig. 3b).

As it may be more clinically relevant to institute pharmacologic intervention *after* the insult has occurred, we also administered temsirolimus 2 hours after LPS injection. Again, we observed increased autophagy in the renal cortex of adult animals that received temsirolimus after LPS (data not shown), which correlated with significantly improved renal function, as evidenced by reduced serum cystatin (542 vs. 1704 ng/mL, p = 0.009) and BUN (63 vs. 113 mg/dL, p = 0.048) concentrations 48 hours after LPS (Fig. 3e). Thus, augmenting autophagy in aged, adult mice produced a temporal pattern of autophagy and AKI similar to that of young mice.

**Figure 4:**
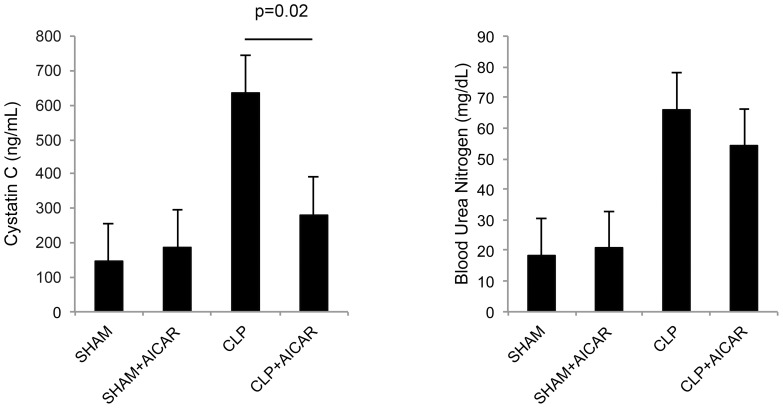
AICAR improves renal function in mice during CLP. Serum cystatin and blood urea nitrogen (BUN) concentrations after CLP or SHAM operation in adult mice receiving either AICAR or diluent control (n = 6 mice per group per timepoint). Data are means ± s.e.m.; rank sum test.

Alternative methods of inducing autophagy yielded similar results of improving renal function during sepsis. AMPK negatively regulates mTOR, and thus AICAR, a biochemical activator of AMPK, has been utilized to induce autophagy [12], [13], [14], [15], [23]. The administration of AICAR augmented autophagy and protected against the development of AKI in the context of CLP sepsis. Young mice receiving AICAR 24 hours prior to CLP had significantly lower cystatin (280 vs. 635 ng/mL, p = 0.02) concentrations at 48 hours, compared with animals that received diluent control prior to CLP (Fig. 4). Mice receiving AICAR alone did not demonstrate derangements in these renal parameters (data not shown).

Our results suggest that aging is characterized by a loss of renal autophagy that may partially explain the increased susceptibility to, and failure to recover from AKI in adult animals during endotoxemia. This is consistent with recent published data suggesting that loss of autophagy is detrimental in the context of experimental nephrotoxic or ischemia/reperfusion (I/R) models. Sudharsan et al demonstrated that pharmacologic inhibition of autophagy with either 3-methyladenine or bafilomycin increased renal proximal tubular cell apoptosis and cell death during cisplatin treatment [24]. Others have shown that proximal tubule-specific autophagy-deficient mice exhibited significantly greater elevation in serum renal function parameters and more severe morphologic derangements in response to cisplatin and I/R injury compared to control mice [6], [25], [26]. Further, Jiang et al reported that concurrent administration of the mTOR inhibitor, rapamycin, activated autophagy and ameliorated cisplatin-induced AKI in 8–10 week old male C57BL/6 mice as determined by lower serum BUN and creatinine, and less prominent histologic damage [26].

However, evidence that harnessing *deficient* autophagic machinery, such as occurs with aging, to treat established AKI in response to a septic stimulus, is lacking. The administration of temsirolimus, even after endotoxemia, effectively increased autophagy in older, adult animals, which corresponded with significantly less derangement in renal function during endotoxemia. This produced a temporal pattern of AKI in adult mice similar to that observed in young animals. However, given the complexity underlying the pathogenesis of septic AKI, autophagy may not be the sole mechanism responsible for the differences observed. Many past investigations have focused on the role of inflammation in promoting AKI, and temsirolimus, like other rapalogues, is not only a potent inducer of autophagy but has anti-inflammatory actions as well. We also noted significantly lower levels of both IL-6 and IL-10 in animals that received temsirolimus before or after LPS (data not shown). However, distinguishing an independent effect of temsirolimus on inflammation rather than on autophagy may be difficult. Recently it has been suggested that autophagy and inflammation are intimately linked. In macrophages a TOR-autophagy spatial coupling compartment (TASCC) augments cellular functions and facilitates the mass synthesis of secretory proteins, such as cytokines [27]. Disruption of the TASCC suppressed the synthesis of IL-6 and IL-8 in macrophages [27]. Thus, the concomitant effects of temsirolimus on autophagy and inflammation may occur through autophagy-dependent cytokine synthesis.

In conclusion, we believe our data support the idea that autophagy has a critical cytoprotective role in AKI – one that also fosters recovery and can be induced pharmacologically in aged animals, which have lost this response. Perhaps the combination of immune response modulation and induction of autophagy provided by temsirolimus is what will ultimately prove beneficial over therapies that target a single mechanistic pathway. The development and implementation of such an agent into clinical practice has the potential to impact the natural course of the disease.
